# The Influence of Cold-Pressed Linseed Cake Supplementation on Fatty-Acid Profile and Fat-Soluble Vitamins of Cows’ Milk in an Organic Production System

**DOI:** 10.3390/ani13101631

**Published:** 2023-05-12

**Authors:** Kamila Puppel, Marcin Gołębiewski, Jan Slósarz, Małgorzata Kunowska-Slósarz, Paweł Solarczyk, Grzegorz Grodkowski, Piotr Kostusiak, Kinga Grodkowska, Beata Madras-Majewska, Tomasz Sakowski

**Affiliations:** 1 Animal Breeding Department, Institute of Animal Science, Warsaw University of Life Sciences, Ciszewskiego 8, 02-786 Warsaw, Poland; 2 Apiculture Division, Institute of Animal Science, Warsaw University of Life Sciences, Nowoursynowska 166, 02-787 Warsaw, Poland; 3 Institute of Genetics and Animal Biotechnology, Polish Academy of Science, Jastrzębiec, Postępu 36A, 05-552 Magdalenka, Poland

**Keywords:** milk, bioactive components, supplementation, linseed cake

## Abstract

**Simple Summary:**

Cow’s milk is a source of many nutrients and bioactive substances that have a positive impact on consumers’ health. The fat fraction is a valuable source of C18:2 *cis*9 *trans*11 and C18:1 *trans*11, as well as the vitamins A, D, E, and K. The concentrations of these substances in milk can vary depending on both environmental and genetic factors. Good-quality milk with high antioxidant potential is obtained from organic production systems where the animals have permanent access to pasture. However, milk from this form of production system shows seasonal fluctuations in the levels of bioactive components, mostly due to the type of feed intake at end of the grazing season. During the winter season, silage and hay are the main feedstuffs for cows; therefore, the level of bioactive substances in the milk decreases. The aim of this study was to determine the effects of using linseed cake supplementation during the winter period on the levels of some bioactive components in the fat fraction of milk from cows kept on an organic farm.

**Abstract:**

The aim of the study was to determine the effect of linseed cake supplementation during the winter period as a factor influencing the level of some bioactive components (milk composition, fatty-acid profile, and fat-soluble vitamins) in the milk fat fraction in cows kept on an organic farm. Forty multiparous (second and third lactation) Holstein–Friesian cows were selected that had 81 ± 12 days in milk and produced 15.08 ± 1.20 kg of milk/day. Two groups were created for the experiment: control (CTL; n = 20) and experimental (LC; n = 20). The experiment was divided into two periods: an initial period lasting 7 days in which the experimental group was habituated to the new supplement in their diet; the proper experimental phase, lasting 6 weeks, in which the cows in the experimental group received an individual daily dose of linseed cake (300 g/day/cow). Linseed cake supplementation had a positive impact on the levels of bioactive components (fatty-acid profile and fat-soluble vitamins) in the milk fat fraction. At the end of the trial, the concentration of C18:2 *cis*9 *trans*11, C18:1 *trans*11, α-retinol, α-tocopherol, and total antioxidant status increased 1.59-, 1.94-, 3.12-, 3.38-, and 3.09-fold, respectively, relative to the control levels. The use of linseed cake in winter on organic farms makes it possible to increase the antioxidant potential of milk, thereby eliminating the disparity in the quality of milk from the summer season compared to the winter season.

## 1. Introduction

In animal husbandry, dietary supplementation is a potential solution to the problem of an unbalanced diet [1]. Ruminant milk fat contains several fatty acids (FA) that have antimutagenic properties, including C4:0, C18:1 *trans*11, and conjugated linoleic acid—C18:2 *cis*9 *trans*11 [2,3]. By using specific diets, it is also possible to modify the composition of the milk or meat obtained from ruminants. Experiments involving the supplementation of cows’ diets that aimed to improve milk composition began appearing as early as the 1970s. Animal diets were usually enriched with unsaturated fatty acids derived mainly from whole seeds and vegetable or fish oils [4,5,6,7]. Linseed supplementation has been reported to result in high concentrations of α-linolenic acid (C18:2 n3; LNA), CLA, and vaccenic acid (C18:1 *trans*11, TVA) in the milk of sheep, cows, and goats [8,9,10]. The main feature of ruminant milk fat is that it contains a high proportion of saturated fatty acids, constituting over 60% of all FAs. Therefore, striving to reduce the SFA levels in cow’s milk and, thus, lowering the atherogenic and thrombogenic indices are among the main goals for modifying cows’ milk fat. Fatty acids that have a chain longer than C18 limit the synthesis of acetic acid and β-hydroxybutyric acid, as well as inhibit acetyl-CoA activity. Therefore, the reduction in SFA content in milk fat derived from fish oil [10] or linseed [6] supplementation has the effect of limiting the de novo synthesis of saturated fatty acids by C18:2 n6 and C18:3 n3, derived from fat additives. Fatty acids, especially polyunsaturated fatty acids, included in the daily diet play an important role as inhibitors of carcinogenic processes. The n3 and n6 PUFA families, as well as C18:2 *cis*9 *trans*11, have potential antioxidant and anticarcinogenic properties, thus allowing the body to maintain a balance between oxidizing substances and antioxidants. Supplementing dairy cow feed with linseed [6], fish oil [10], or a mixture of fish oil and linseed [5] increases the activity of elongase and Δ-9 desaturase enzymes, which is consequently associated with an increase in the C18:2 n6, C18:3 n3, C18:1 *tran*s11, C20:5 n3 (DPA), C22:6 n3 (DHA), and C18:2 *cis*9 *trans*11 concentrations in milk fat. Supplementing the diet of cows with fish oil and linseed significantly influences antioxidant capacity. Additionally, the response of multiparous and primiparous cows to the introduced supplement is noticeably different. Thus, it may be concluded that the age of cows significantly influences antioxidant capacity [5]. The variety of linseed used significantly influences the lipid fraction level and the basic chemical composition of cow’s milk. The linseed variety should, therefore, be taken into consideration in subsequent experiments, in addition to the quantity and physical form of the linseed [6]. Feeding animals these ingredients makes it possible to ensure that they have an adequate supply of fat, and that their essential fatty acid (EFA) demands are met. In particular, it is important to supplement the animals’ diets with adequate amounts of α-linolenic and linoleic acids [11]. Additionally, Neofytou et al. [12] reported that including ensiled olive cakes in cows’ diets improved the lipid profile of bovine milk in a way that was beneficial for human health.

The literature provides information about the influence of linseed meal on the quality of milk obtained from cows fed with it. Kudrna and Marounek [13] conducted a comparative study on the addition of palm oil, extracted linseed, and whole sunflower seeds to the diets of cows fed on a total mixed ration (TMR) regimen. In their results, the authors observed the highest CLA, linoleic acid, and linolenic acid content—in relation to other research groups—in milk obtained from cows in the group supplemented with the linseed meal. Correddu et al. [14] reported that grape seed and linseed could be useful for increasing FA concentrations and, thus, producing potential health benefits, especially when these ingredients are included in diets in combination.

Linseed cake is a byproduct of cold-pressed linseed oil production, and it is a valuable additive because of its protein and fat content. An interesting feature of the oil cake is the presence of a mucilage coating on the seeds, which has a protective effect on the digestive tract of animals. Swistowska et al. [15], in their research, dealt with determining the influence of linseed cake supplementation on the performance of sport horses. Their research showed that this additive increased the amount of energy delivered in the ration, allowing the ration to be reduced without affecting the horses’ health. The authors also emphasized that the cake contained protein that was very easily digested by animals. Its digestibility level ranged from 85% to 90%, and they pointed out that linseed cake is also a source of tryptophan—an essential, exogenous amino acid. In the course of research, it has been proven that linseed cake has a positive influence not only on the animals fed with it, but also on the products obtained from it. Nudda et al. [16] showed that, after supplementation, there was a slight decrease in milk yield in their test groups relative to the control; however, what is most important is that the authors noted a definite increase in valuable fatty-acid content, i.e., DHA, EPA, CLA, and TVA. In a study conducted by Horky et al. [17] on cows kept on organic farms, the authors showed that supplementation with linseed cake additionally increased the levels of, among others, proline, alanine, and valine in the milk.

Puppel et al. [6] showed that the variety of linseed shapes the level of bioactive components of both the fat and protein fractions. Therefore, when choosing linseed, one should consider not only their form (crushed, rolled, or whole) and quantity, but also the variety of seeds. However, the study Puppel et al. [6] involved an intensive production system. Therefore, there is a need to verify the possibility of using supplements rich in n3 in an organic production system. All these data allow us to hypothesize that linseed cake supplementation would be an ideal way to supplement cows’ diets with essential fatty acids and would be a method for maintaining the milk’s high antioxidant quality during the winter season. Good-quality milk with high antioxidant potential is obtained from cows kept on organic farms where the animals have permanent access to pasture. During the winter season, silage and hay are the main sources of nutrition for cows; therefore, the level of bioactive substances in the milk decreases. The aim of this study was to determine the effect of using linseed cake supplementation during the winter period as a factor that influences the level of some bioactive components in milk fat fraction in cows kept on an organic farm.

## 2. Materials and Methods

All cows were handled in accordance with the regulations of the Polish Council on Animal Care, and all procedures were reviewed and approved by the Second Ethics Committee for Animal Experimentation in Warsaw (approval number: WAWA2/086/2018). During the experiment, the cows were under veterinary control.

The experiment was performed at the Juchowo certified organic biodynamic farm in Poland. All measures and activities used in a biodynamic farm are subject to evaluation according to holistic principles. It is important not only to balance the material needs of the system in question, but also to balance the loss of life forces. It is of great importance to pay conscious attention to the details involved in the production, storage, and use of biodynamic preparations. Cows are bred according to the principles of breeding for longevity. The basis of this type of breeding is the selection of animals for longevity, which is reflected in the numerous calving of calves, with increasing milk yield to the age of 7–8 years and a high overall milk yield over the course of their lives. Forty multiparous (second and third lactation) Holstein–Friesian cows were selected that had 81 ± 12 DIM (days in milk) (mean ± SD) and produced 15.08 ± 1.20 kg of milk/day. Average milk yield was about 6500 L/cow/year. The body condition score (BCS) was assessed using the BCS-5 method described by Edmonson et al. [18]; the average BCS for the cows was 3.0–3.4. During the study, the cows were under veterinary care and did not show any disorders or diseases. In addition, the cytological quality of the milk expressed in the milk somatic cell count (SCC) was in the range of 85,000–180,000/mL. Therefore, it can be concluded that there were no mastitic changes in the cows participating in the experiment.

Two groups were created for the experiment: control (CTL; n = 20) and experimental (LC; n = 20). The experiment was divided into two periods: an initial period lasting 7 days, in which the cows in the experimental group were habituated to the new supplement in their diet; the proper experimental phase, lasting 6 weeks, in which the cows in the experimental group received an individual daily dose of linseed cake (300 g/day/cow). The supplement was administered every day at the same time (in the morning) after milking. The linseed cake used in this experiment contained the following FAs (in g/100 g of fat): C12:0–0.21, C14:0–0.22, C16:0–6.42, C16:1–0.41, C18:0–4.71, C18:1–20.98, C18:2 n-6-14.13, and C18:3 n-3-56.12. The control diet (Table 1) contained the following FAs (in g/100 g of fat): C14:0–0.92, C16:0–19.12, C16:1–0.63, C18:0–18.73, C18:1–22.08, C18:2 n-6-3.14, and C18:3 n-3-1.85.

Representative milk samples were collected from each cow during milking by means of a milk auto-sampler situated in the milking parlor. The cows were milked daily at 5:30 a.m. and 5:30 p.m., and the milk yield was recorded at each milking. Milk samples were collected twice—on the first day of the experiment (CTL1, LS1) and again after 6 weeks of supplementation (CTL2, LS2). The combined milk from the morning and evening milking was placed in sterile bottles and preserved with Mlekostat CC.

### 2.1. Chemical Analysis

The basic chemical composition of the milk (fat, total protein, lactose, casein, and urea) was determined by infrared spectrophotometry using a MilkoScan FT-120 analyzer.

Extraction of the fat was performed according to the Röse–Gottlieb procedure [19]. The concentrations of fat-soluble vitamins and β-carotene (BK) were determined using an Agilent 1100 Series RP-HPLC (Agilent Technologies, Waldbronn, Germany) according to the methodology described by Puppel et al. [20]. Separations were performed at ambient temperature using a solvent gradient on a C18 300A Jupiter column (Phenomenex, Torrance, CA, USA) and an Agilent UV detector. The chromatographic conditions were as follows: solvent A consisted of methanol (Merck, Darmstadt, Germany) and water (Sigma-Aldrich, St Louis, MO, USA) at a ratio of 100:900 (*v*/*v*); solvent B consisted of water and methanol at a ratio of 900:100 (*v*/*v*). The total run time was 7 min, the flow rate was 1.2 mL/min, and the detection wavelength was 280 nm. The injection volume of the final solution was 25 μL. All samples were analyzed in duplicate. The identification of peaks as selected vitamins was confirmed by comparison to the standards (Sigma-Aldrich, St Louis, MO, USA).

Fatty-acid methylation was carried out using the *trans-*esterification method PN-EN ISO 5509:2000 [21]. The concentrations of fatty acids were determined using an Agilent 7890 GC gas chromatograph (Agilent Technologies, Waldbronn, Germany), a flame ionization detector, and a Varian Select FAME column (Varian, Agilent Technologies, Waldbronn, Germany) according to the methodology described by Puppel et al. [11]. The separation was performed at the preprogrammed temperatures of 130 °C for 1 min, 130–170 °C at 6.5 °C/min, 170–215 °C at 2.75 °C/min, 215 °C for 12 min, 215–230 °C at 2 °C/min, and 230 °C for 3 min. Helium, at a flow rate of 25 cm/s and a constant pressure, was used as the carrier gas; the injector’s temperature was 240 °C, and the detector’s temperature was 300 °C. The identification of acids was carried out against the following standards: PUFA no. 1, Lot LB 75,066; PUFA no. 2, Lot LB 83,491; FAME Mix RM-6, Lot LB 68,242; Supelco 37 Comp. FAME Mix, Lot LB 68,887 (Supelco, Bellefonte, PA, USA).

The total antioxidant potential (TAS) was determined using the RANDOX application; incubation of ABTS^®^ with peroxidase (methemoglobin) leads to the formation of the radical cation ABTS++. This substance is blue-green and can be detected at 600 nm. Antioxidants present in the sample reduce the formation of the blue-green color in proportion to their concentration. U/L defines the TAS concentration as follows, where HX-Fe(III) is methemoglobin, X-[FeIV = 0] is feroglobin, and ABTS^®^ is 2,2-azino-di [3-ethylbenzothiazolinosulfonate] (RANDOX materials):

HX-Fe^III^ + H_2_O_2_ → X-[Fe^IV^ = 0] + H_2_O,

ABTS^®^ + X-[Fe^IV^ = 0] → ABTS^®+^ + HX-Fe^III^.

### 2.2. Statistical Analysis

The data obtained were statistically processed using multivariate analysis of variance by the least squares method using SPSS 22 software (Armonk, NY, USA) [22].

The model used for milk composition was

*Y_ijkl_* = *μ* + *A_i_* + *B_j_* + (*A_i_* × *B_j_*) + *e_ijkl_*,

where *Y_ijkl_* is the dependent variable, *μ* is the general mean, *A_i_* is the treatment effect (where *i* = 1 or 2, in which 1 refers to the control, and 2 refers to LC), *B_j_* is the week effect (where *j* = 1 − 2, in which 1 refers to the control collecting, and 2 refers to 6 weeks of experiment), (*A_i_* × *B_j_*) is the fixed interaction effect between treatment and week, and *e_ijkl_* is the random error.

Only those interactions between factors whose effect was statistically significant (*p* ≤ 0.01 or *p* ≤ 0.05) were included in the study, as determined after preliminary statistical analyses. Data were presented as the least squares mean (LSM) with the standard error of the mean (SEM). For multiple comparisons, the Duncan test was used.

## 3. Results and Discussion

At the beginning of the experiment, the gross composition of the milk from both the CTL and the LC groups were very similar to each other and were within normal limits (Table 2). In the CTL group, the average casein and lactose content decreased after the 6 week supplementation period by 0.09% and 0.1%, respectively. However, this trend did not apply to the development of protein levels, which increased by 0.04%. Thus, in the present study, the supplementation of linseed cake affected the protein content, as found by other authors [23,24]. Although Castellani et al. [25] observed that milk protein content was influenced by adding dried olive cake to the cows’ diets, the authors attributed these unexpected results to a difference in the forage-concentrate ratio of the diets.

The literature data concerning the influence of supplementation with additives containing vegetable oils on the fat content in milk are not unequivocal, and the obtained results do not show the same relations. Jóźwik et al. [26], in their study comparing the effect of rapeseed cake and linseed cake on milk obtained from cows, did not observe any effect from these two additives on milk fat content; this is similar to the results of the present study. On the other hand, when meal extracted from linseed was added to the diet of animals in an experiment by Kudrna and Marounek [13], an increase was shown in the fat content of the milk from the animals involved in the experiment.

High concentrations of saturated fatty acids (SFA) are characteristic of cows’ milk; more than half of all fatty acids have this configuration. Fatty acids with 4–14 carbon atoms are formed in the mammary gland as a result of the synthesis of volatile fatty acids (from acetates and hydroxybutyrates). The C16:0 acid is 50% synthesized de novo from the same precursors, and the remaining 50% is synthesized from blood lipids. The decreased proportion of acetic acid in relation to propionic acid reduces the de novo synthesis of fatty acids. Fatty acids with chain lengths >C18 delivered in cows’ feed ratio reduce the production of acetic acid, β-hydroxybutyric acid, and the activity of acetyl-CoA, thereby lowering the concentration of saturated fatty acids. In light of the conducted nutritional studies, SFAs are an etiological factor for many diseases, including cardiovascular diseases. C12:0, C14:0, and C16:0 acids have atherogenic properties, while C14:0, C16:0, and C18:0 acids are characterized by thrombogenic properties [27]. Therefore, reducing the level of SFAs in cows’ milk should be one of the main goals for modifying cows’ milk fat. Supplementing the LC cows progressively decreased the milk saturated fatty-acid concentration due to the decreased secretion of 6–16-carbon FAs (Table 3). The exception was butyric acid, whose levels were elevated after the 6 week supplementation period. Similar correlations have been demonstrated by other research teams; for example, in a small ruminant experiment by Jóźwik et al. [23], after supplementing goats with linseed cake for 4 weeks, the authors also observed a decrease in SFAs in total fatty acids.

Interestingly, in the present study, the levels of the analyzed acids 6 weeks after the first collection were elevated for the CTL group in contrast to the LC group. The only exception to this was hexanoic acid, whose levels decreased slightly. The observed relation resulting from the lowering of the SFA content in the milk from the LC group proves that the applied supplementation had a significant influence on limiting the level of fatty-acid saturation. It should be emphasized that, in the case of two acids—palmitic and myristic, a statistically significant decrease in concentration occurred after the 6 week supplementation period: from 40.403 g/100 g of fat to 37.974 g/100 g for palmitic acid, and from 11.602 g/100 g of fat to 9.133 g/100 g for myristic acid (Table 3). Increases in the availability of ≥16-carbon-chain FAs are known to inhibit mammary acetyl-CoA carboxylase activity [28]. A similar relationship was shown by Kudrna and Marounek [13] as an effect of supplementation with linseed post-extraction meal. This supplementation also caused a decrease in the level of saturated acids, including palmitic acid, in the cows’ milk. It is also worth mentioning that myristic acid has a negative impact on the cardiovascular system; therefore, lowering its concentration in milk is beneficial from the point of view of consumer health.

In the present study, the reduction in SFAs was accompanied by increased total monounsaturated fatty acid (MUFA) content in the supplemented LC group. It is likely that the MUFAs in the feed were transferred to the milk’s FA content through mammary uptake from the plasma dietary FAs, contributing to the higher MUFA content in the milk [29]. In the case of the acids belonging to the MUFA group, the levels of C12:1 *cis*9, C14:1 *cis*9, C16:1 *cis*9, C17:1 *cis*10, vaccenic (C18:1 *trans*11), and oleic (C18:1 *cis*9) acids were analyzed. It should be noted that, after 6 weeks, in both the CTL and the LC groups, the levels of individual monounsaturated fatty acids changed (Table 3). The research showed that the applied supplementation had a statistically significant influence on the level of monounsaturated fatty acids in the milk fat.

In the case of the first two MUFAs, C12:1 *cis*9 and C14:1 *cis*9, a decrease in concentration was demonstrated in both the research and the control groups. This suggests that the linseed cake supplementation had no effect on the levels of these two fatty acids. The mean level of palmitic oleic acid also changed during the experiment, in both the LC and the CTL groups. For the CTL group, the mean value after 6 weeks decreased, while the level increased slightly in the LC group (Table 3). The effect of LC on the milk’s 18-carbon FA composition can be explained by differences in the profiles of the intermediates formed during the biohydrogenation of C18:1 *cis*9 and C 18:3 n3 in the rumen [29]. The greatest changes were found when analyzing the levels of bioactive vaccenic acid and oleic acid (Table 3). Loor et al. [30] reported that higher inputs of UFAs resulted in the accumulation of *trans*-18:1. Vaccenic acid (C18:1 *trans*11) is a desirable FA that flows from the rumen, because it can be used as substrate for producing CLA *cis*9 *trans*11 in animal tissue via Δ9-desaturase [31]. In contrast to linoleic acid, TVA is the product of the hydrogenation of linolenic acid, which accounts for the significant content increase in the milk [32]. Vaccenic acid levels in the LC group more than doubled after 6 weeks of supplementation. The average level increased by as much as 1.815 g/100 g of fat. The results of our own research concerning C18:1 *trans*11 under the influence of linseed cake supplementation are in accordance with data obtained by other research teams, e.g., Jóźwik et al. [22] and Nudda et al. [16]. To sum up, after 6 weeks of supplementation, the concentrations of C16:1 *cis*9, C18:1 *trans*11, and C18:1 *cis*9 increased by 7%, 59%, and 10%, respectively, relative to the control levels.

In the present study, the mean level of each of the analyzed PUFAs increased after applying 6 weeks of supplementation with linseed cake (Table 3). Fatty acids determine not only the quality of milk but also its technological quality. Fatty acids belonging to the n3 family (C18:3 n3, C20:5 n3, and C22:6 n3) and the n6 family (C18:2 n6, C18:3 n6, amd C20:3 n6), as well as C18:2 cis9 trans11, are characterized by potential antioxidant and anticarcinogenic properties; thus, they maintain a balance between oxidizing substances and antioxidants in the human body. It is, therefore, important to study the sources of FA variability in milk, which include the maintenance system, type of breeding, and system of production. The synthesis of fatty acids is a cycle of eight enzymes: acyl-CoA synthase, acyl CoA carboxylase, acyltransferase, ketoacyl reductase, redoxylase dehydratase, enoyl reductase, thioesterase, and the acyl carrier [33]. Elongase enzymes lengthen carbon chains, and desaturases generate additional double bonds, resulting in the formation of polyunsaturated fatty acids with a chain length of ≥C20. C18:2 n6 and C18:3 n3 acids give rise to the n6 and n3 fatty acid families, respectively. Because of the changes that occur in the plasma reticulum, C18:3 n6 acid is formed from C18:2 n6 acid, which is extended to C20:3 n-6, and then to C20:4 n6. In a similar process C20:5 n3 is produced from C18:3 n3, which is then converted by the Δ-6 desaturase enzyme into C20:2 n6. C20:4 n6 is formed as a result of the desaturation and elongation of C18:2 n6, and C20:5 n3 is formed as a result of the desaturation and elongation of C18:3 n3. In both cases, the Δ-6 desaturase enzyme plays a key role [34]. C20:5 n3 is a competence antagonist relative to C20:4 n-6. Therefore, any feed ratio for cows that contains a significant amount of C18:2 n3 inhibits the synthesis of C20:4 n-6, as well as causes the transfer of elongase and desaturase enzymes to the PUFA n3 family of fatty acids [35]. Lerch et al. [36] reported that the addition of the unsaturated fatty acids contained in oilseeds attenuated the concentration of saturated fatty acids in milk, which led to an increase in the proportion of fatty acids, among others: C18:1 *cis*9, C18:3 n3, and CLA *cis*9 *trans*11. This process was also observed in the present study. In our experiment, the average level of linoleic and linolenic acids, of which linseed cake is a source, increased significantly. For linoleic acid, the value at the end of the experiment was 148% of the initial value (Table 3). In a study by Jóźwik et al. [25], the linoleic acid content in milk from supplemented cows increased by 100%. As far as linolenic acid is concerned, the average content increased from 0.444 g/100 g of fat to 0.715 g/100 g of fat.

Previous studies [5,6,10] have shown, that feeding lipid sources to animals can modulate the concentration of CLA *cis*9 *trans*11 in milk fat, as this isomer is associated with anticarcinogenic properties [37], in addition to enhancing immune function [38,39,40] and reducing inflammation, asthma [41], and arteriosclerosis [42]. With the inclusion of linseed cake in the animals’ diets, it was possible to increase the isomer’s level from 0.354 g/100 g of fat to 0.884 g/100 g of fat (Table 3). Flowers et al. [43], after including linseed oil in the diets of grazing cows, showed there was a correlation between the amount of oil in the diet and CLA content, the level of which increased linearly. The authors reported that a marked increase in TVA levels in milk, and its subsequent conversion to *cis*9 *trans*11 CLA by desaturase in the mammary gland, may explain the linear increase in milk. In another experiment performed by Flowers et al. [43], the control diet’s acid level was 1.12 g/100 g, while it reached 1.65 g/100 g in the diet that included 510 g of linseed oil. A significant increase in CLA content in the milk of cows has also been noted when other sources of linseed oil are used. Kudrna and Marounek [13], in their work, also reported a significant increase in the level of CLA isomers after including linseed extract meal in the diet of animals. In their experiment, the authors compared the effects of two other supplements, whole sunflower seeds and palm oil, but it was the milk obtained from cows fed the linseed supplement that had the highest CLA content in its composition, reaching a value of 1.40 g/100 g. Baumgard et al. [44] reported that CLA *trans*10 *cis*12 is known to inhibit milk fat synthesis in cows. However, a lack of increase in these FAs in milk fat tends to suggest that they had no major role in contributing to decreases in de novo mammary FA synthesis in cows fed LC in the present study (Table 3).

In the case of another valuable acid, EPA, an increase was also noted in the average level in the milk of the studied animals (Table 3); the value recorded in the experimental group at the end of the experiment was 146% of the initial value. In the control group, the level remained similar throughout the experiment and increased by only 0.008 g/100 g of fat. A slightly smaller increase was observed in the LC group for DHA. Its mean level after the supplementation period increased by 0.022 g/100 g (Table 3). A slight increase in DHA concentration was also reported by Nudda et al. [16] when linseed cake was added to goats’ diets. In their study, the levels of this acid increased from 0.039 mg/100 mg of total fatty acids to 0.045 mg using a diet containing 5% linseed cake.

To sum up, after 6 weeks of supplementation, the concentrations of C18:3 n6, C18:3 n3, C18:2 *cis*9 *trans*11, C22:5 n3, and C22:6 n3 increased by 275%, 132%, 194%, 30%, and 25%, respectively, relative to the control levels.

Oxidative processes have several implications for milk and other dairy products, such as reduced shelf-life, diminished flavor, and deterioration in nutritional quality [45]. However, protein oxidation occurs independently of lipid oxidation. Havemose et al. [46] reported that a higher concentration of antioxidant is able to extend the lag phase of protein oxidation and delay the formation of dityrosine. To improve the nutritional and health characteristics of milk, the best option would be to lower cholesterol content and increase its antioxidant protection. Therefore, to improve the nutritional and functional properties of milk, it is necessary to increase the levels of bioactive ingredients that have antioxidant properties.

Our study showed that the applied linseed cake supplementation also had a statistically significant influence on the level of fat-soluble vitamins and β-carotene. The level of beta-carotene achieved in the LC group’s milk is noteworthy. After the supplementation period, it increased more than fivefold to a value that was 569% of the initial value (Table 4). The canned feed that is used on organic farms during the winter season are poor in carotenoids, which may have contributed to lower beta-carotene levels [47] observed in the control group. Supplementation with linseed cake was able to negate this phenomenon. A similar result was obtained for vitamin E content (Table 4). Its level, in relation to the content at the beginning of the experiment, increased more than fourfold. A similar change was also observed for the levels of the vitamins D and K, which more than tripled compared to the initial content (Table 4).

An equal result was achieved in the case of vitamin A, whose level rose to 2.525 mg/L of milk, more than three times the average initial value (Table 4). This was probably related to the increased conversion of *β*-carotene to *α*-retinol [48]. At the same time, in the control group, only insignificant variations in the levels of the vitamins being studied were noted. In the present experiment, the inclusion of linseed cake in the diet of cows significantly increased the content of fat-soluble vitamins. Milk from cows grazing on pasture manifest a higher fat-soluble vitamin content relative to animals that do not have access to pasture forage during the winter season [49]. In a study by Radkowska [50], the average vitamin E content in the milk of animals feeding on pasture during the summer season was 1.185 µg/L, while that of vitamin A was 0.485 µg/L. The results achieved in this study far exceeded those values. With the use of linseed cake supplementation, it is possible to significantly increase the vitamin content of cows’ milk and make it more attractive to consumers.

Puppel et al. [7] reported that DAP and TAS values were at their highest levels when pasture herbage was dominant in the feeding treatment. The study showed a significant effect of the supplementation used on the formation of TAS levels (Table 4). However, it has been shown that the addition of linseed cake increased the degree of antioxidant protection. Therefore, winter nutrition in organic production systems should also include energy additives.

To sum up, after 6 weeks of supplementation, the concentrations of beta-carotene, *α*-retinol, *α*-tocopherol, and TAS increased by 550%, 312%, 338%, and 309%, respectively, relative to the control levels.

## 4. Conclusions

Linseed cake supplementation had a positive impact on the levels of some bioactive components (milk composition, fatty-acid profile, and fat-soluble vitamins) in the milk fat fraction. After 6 weeks of supplementation, the concentrations of C18:1 *trans*11, C18:2 *cis*9 *trans*11, α-retinol, α-tocopherol, and total antioxidant status increased 1.59-, 1.94-, 3.12-, 3.38-, and 3.09-fold, respectively, relative to the control levels.

Additionally, as production waste from the cold-pressing of oil, using linseed cake is both economical and environmentally friendly. The use of linseed cake in winter on organic farms makes it possible to provide animals with the same necessary nutrients that pasture grass provides during the summer, thus enriching their milk with the valuable bioactive compounds, C18:1 *trans*11, C18:2 *cis*9 *trans*11, α-retinol, and α-tocopherol.

## Figures and Tables

**Table 1:** Ingredients and chemical composition of the diets.

Composition	Treatment	
	Control Diet	Experimental Diet
**Ingredient (kg/day)**		
Grass silage	24.00	24.00
Alfalfa silage	71.00	71.00
Corn silage	3.50	3.50
Pasture ground chalk	0.10	0.10
Vitamin mix	0.14	0.14
Salt	0.05	0.05
Magnesium oxide	0.05	0.05
Linseed cake	0.00	0.30
**Chemical composition**		
Dry matter, %	57.50	58.20
Ash, % of DM	4.20	4.45
Crude protein, % of DM	7.50	7.75
Acid detergent fiber, % of DM	27.90	28.70
Neutral detergent fiber, % of DM	33.70	34.60
Calcium, % of DM	0.90	1.01
Phosphorus, % of DM	0.50	0.60
Crude fiber, % of DM	4.36	4.56
UFL per kg of DM	1.10	1.16

**Table 2:** Effect of linseed cake supplementation on the milk’s gross composition [%]. CTL—control group; LC—group receiving linseed cake (300 g/day/cow); CTL1—after 6 weeks; LC1—after 6 weeks of supplementation; CTL2—after 6 weeks; LC2—after 6 weeks of supplementation. Data are presented as the least squares mean with the standard error of the mean; (*A_i_* × *B_j_*) is the fixed interaction effect between treatment and week; NS—not significant.

Milk’s Gross Composition [%]	Diet				SEM	*p*-Value	Interaction, *A_i_* × *B_j_*
	Control (CTL)		Experimental (LC)				
	CTL1	CTL2	LC1	LC2			
Casein	2.63	2.52	2.58	2.50	0.325	*p* ≤ 0.05	*p* ≤ 0.05
Protein	3.01	3.05	2.96	3.03	0.214	*p* ≤ 0.05	*p* ≤ 0.05
Fat	5.98	4.06	5.64	4.08	0.229	*p* ≤ 0.01	*p* ≤ 0.05
Lactose	4.71	4.61	4.73	4.62	0.347	NS	NS

**Table 3:** Effect of linseed cake supplementation on the level of selected fatty acids [g/100 g fat]. CTL—control group; LC—group receiving linseed cake (300 g/day/cow); CTL1—after 6 weeks; LC1—after 6 weeks of supplementation; CTL2—after 6 weeks; LC2—after 6 weeks of supplementation. Data are presented as the least squares mean with the standard error of the mean; (*A_i_* × *B_j_*) is the fixed interaction effect between treatment and week; NS—not significant.

Selected Fatty Acids [g/100 g fat]	Diet				SEM	*p*-Value	Interaction, *A_i_* × *B_j_*
	Control (CTL)		Experimental (LC)				
	CTL1	CTL2	LC1	LC2			
C4:0	1.841	1.954	2.117	2.135	0.115	*p* ≤ 0.01	*p* ≤ 0.01
C6:0	1.837	1.816	1.821	1.690	0.187	*p* ≤ 0.01	*p* ≤ 0.01
C8:0	1.116	1.126	1.085	1.079	0.203	*p* ≤ 0.01	*p* ≤ 0.05
C10:0	2.828	2.873	2.905	2.793	0.147	*p* ≤ 0.05	*p* ≤ 0.05
C12:0	3.457	3.496	3.583	3.358	0.298	NS	NS
C15:0	1.084	1.091	1.131	1.034	0.085	NS	NS
C14:0	11.584	11.715	11.602	9.133	0.318	*p* ≤ 0.01	*p* ≤ 0.01
C16:0	39.954	40.33	40.403	37.974	0.412	*p* ≤ 0.01	*p* ≤ 0.01
C12:1 *cis*9	0.152	0.146	0.131	0.128	0.035	NS	NS
C14:1 *cis*9	0.878	0.870	0.933	0.821	0.082	NS	NS
C16:1 *cis*9	1.864	1.791	1.814	1.934	0.241	*p* ≤ 0.05	*p* ≤ 0.05
C17:1 *cis*10	0.293	0.265	0.257	0.206	0.044	*p* ≤ 0.05	*p* ≤ 0.05
C18:1 *trans*11	2.063	2.298	1.844	3.658	0.265	*p* ≤ 0.01	*p* ≤ 0.01
C18:1 *cis*9	18.005	18.648	15.353	20.726	0.412	*p* ≤ 0.01	*p* ≤ 0.01
C18:2 n6	2.21	2.28	1.952	2.902	0.241	*p* ≤ 0.01	*p* ≤ 0.01
C18:3 n6	0.116	0.12	0.103	0.331	0.023	*p* ≤ 0.01	*p* ≤ 0.01
C18:3 n3	0.488	0.541	0.444	0.715	0.047	*p* ≤ 0.01	*p* ≤ 0.01
C18:2 *cis*9 *trans*11	0.369	0.438	0.333	0.851	0.061	*p* ≤ 0.01	*p* ≤ 0.01
C18:2 *trans*10 *cis*12	0.022	0.026	0.024	0.027	0.002	*p* ≤ 0.05	*p* ≤ 0.05
C20:4 n6	0.127	0.133	0.14	0.152	0.012	NS	NS
C20:5 n3	0.091	0.099	0.078	0.114	0.011	*p* ≤ 0.01	*p* ≤ 0.01
C22:5 n3	0.062	0.068	0.067	0.089	0.008	*p* ≤ 0.01	*p* ≤ 0.01
C22:6 n3	0.018	0.023	0.019	0.031	0.016	*p* ≤ 0.01	*p* ≤ 0.01

**Table 4:** Effect of linseed cake supplementation on fat-soluble vitamins, β-carotene, and TAS. CTL—control group; LC—group receiving linseed cake (300 g/day/cow); CTL1—after 6 weeks; LC1—after 6 weeks of supplementation; CTL2—after 6 weeks; LC2—after 6 weeks of supplementation. Data are presented as the least squares mean with the standard error of the mean; (*A_i_* × *B_j_*) is the fixed interaction effect between treatment and week.

Fat-Soluble Vitamins, β-Carotene and TAS	Diet				SEM	*p*-Value	Interaction, *A_i_* × *B_j_*
	Control (CTL)		Experimental (LC)				
	CTL1	CTL2	LC1	LC2			
β-carotene [mg/L]	0.254	0.216	0.210	1.196	0.111	*p* ≤ 0.01	*p* ≤ 0.01
Vitamin A [mg/L]	0.716	0.808	0.697	2.525	0.068	*p* ≤ 0.01	*p* ≤ 0.01
Vitamin E [mg/L]	0.779	0.976	0.803	3.305	0.071	*p* ≤ 0.01	*p* ≤ 0.01
Vitamin K2 [µg/L]	2.692	2.575	2.256	6.864	0.130	*p* ≤ 0.01	*p* ≤ 0.01
Vitamin D3 [µg/L]	5.81	5.651	6.486	20.071	0.214	*p* ≤ 0.01	*p* ≤ 0.01
TAS [mmol/L]	1.115	1.182	1.061	3.656	0.265	*p* ≤ 0.01	*p* ≤ 0.01

## Data Availability

All data generated or analyzed during the study are included in this published article. The datasets used and/or analyzed in the current study are available from the corresponding author on reasonable request.
